# A nuclear transport-related gene signature combined with IDH mutation and 1p/19q codeletion better predicts the prognosis of glioma patients

**DOI:** 10.1186/s12885-020-07552-3

**Published:** 2020-11-09

**Authors:** Zheng Zhu, Yang Lan, Lihong Wang, Jia Ge, Jiao Wang, Feng Liu, Zhicheng He, Hua Zhang, Min Luo, Dandan Lin, Yaoyao Tan, Yuanyuan Xu, Tao Luo

**Affiliations:** 1 Institute of Pathology and Southwest Cancer Center, Southwest Hospital, Third Military Medical University (Army Medical University) and Key Laboratory of Tumor Immunopathology, Ministry of Education of China, Chongqing, 400038 China; 2 PLA Rocket Force Characteristic Medical Center, Beijing, 100088 China

**Keywords:** Nuclear transport, TCGA, CGGA, Gliomas, Prognosis, Gene signature, Classification

## Abstract

**Background:**

The nuclear transport system has been proposed to be indispensable for cell proliferation and invasion in cancers. Prognostic biomarkers and molecular targets in nuclear transport systems have been developed. However, no systematic analysis of genes related to nuclear transport in gliomas has been performed. An integrated prognostic classification involving mutation and nuclear transport gene signatures has not yet been explored.

**Methods:**

In the present study, we analyzed gliomas from a training cohort (TCGA dataset, *n* = 660) and validation cohort (CGGA dataset, *n* = 668) to develop a prognostic nuclear transport gene signature and generate an integrated classification system. Gene set enrichment analysis (GSEA) showed that glioblastoma (GBM) was mainly enriched in nuclear transport progress compared to lower-grade glioma (LGG). Then, we developed a nuclear transport risk score (NTRS) for gliomas with a training cohort. NTRS was significantly correlated with clinical and genetic characteristics, including grade, age, histology, IDH status and 1p/19q codeletion, in the training and validation cohorts.

**Results:**

Survival analysis revealed that patients with a higher NTRS exhibited shorter overall survival. NTRS showed better prognostic value compared to classical molecular markers, including IDH status and 1p/19q codeletion. Furthermore, univariate and multivariate analyses indicated that NTRS was an independent prognostic factor for gliomas. Enrichment map and Gene Ontology analysis demonstrated that signaling pathways related to the cell cycle were enriched in the NTRS^High^ group. Subgroup survival analysis revealed that NTRS could differentiate the outcomes of low- and high-risk patients with wild-type IDH or mutant IDH and 1p/19q non-codeletion.

**Conclusions:**

NTRS is associated with poor outcomes and could be an independent prognostic marker in diffuse gliomas. Prognostic classification combined with IDH mutation, 1p/19q codeletion and NTRS could better predict the survival of glioma patients.

**Supplementary Information:**

The online version contains supplementary material available at 10.1186/s12885-020-07552-3.

## Background

Eukaryotic cells are divided into the nucleus and cytoplasm by the nuclear membrane. The movement of macromolecules between the nucleus and the cytoplasm, mostly including proteins and RNAs, occurs via the nuclear transport system [1]. The nuclear transport system includes three main components: the nuclear pore complex (NPC), RanGTPase and the nuclear transport receptor (NTR) [2–4]. It has been reported that the nuclear transport system plays an indispensable role in cancer development and metastasis [5, 6]. Targeting the nuclear transport system could be a promising therapeutic approach [7, 8]. However, a single molecule cannot represent the overall activity of the system, and a systemic analysis of nuclear transport and its prognostic value in cancer involving an expression profile is lacking.

Gliomas are the most common primary tumors of the central nervous system and are classified by histologic and genomic phenotype [9, 10]. IDH mutation are common in glioma, acute myeloid leukemia, chondrosarcoma and cholangiocarcinoma. The mutant IDH acquire the activity that converting α-ketoglutarate (α-KG) to D-2-hydroxyglutarate (D2HG) which inhibits a class of α-KG-dependent enzymes involved in epigenetic regulation, collagen synthesis, and cell signaling [11].. 1p/19q codeletion even trump the histological phenotype for oligodendroglioma [9]. In fact, it is not only genomic characteristics such as IDH mutation and 1p/19q codeletion but also transcriptomic and epigenetic characteristics that can be used as biomarkers of molecular classification [12, 13]. Many models of gene signatures based on RNA-seq data can predict prognosis and be employed as an independent prognostic factor [14–16]. However, integrated prognostic classification with classical molecular biomarkers requires further study.

In this study, using RNA-seq data from TCGA as a training cohort and data from CGGA as a validation cohort, we established a nuclear transport risk score (NTRS) and tested the correlations between NTRS and clinicopathologic characteristics. We found that NTRS was an independent biomarker of prognosis and was associated with cell cycle-related pathways. Finally, combined with IDH mutation and 1p/19q codeletion, the value of NTRS in prognostic classification was validated. Taken together, our results indicated that the nuclear transport-related gene signature was strongly associated with poor outcomes and could serve as a novel biomarker for prognostic classification in diffuse gliomas.

## Methods

### Data source

The data from the TCGA training set included RNA-seq data and clinical data from patients (*n* = 660) with LGG and GBM from cBioPortal (http://www.cbioportal.org) [12, 13]. The glioma patients included in the validation set (*n* = 668) came from CGGA (http://www.cgga.org.cn/index.jsp) [17]. The microarray data of Rembrandt, Grevendeel and Kamoun cohorts were obtained from Gliovis (http://gliovis.bioinfo.cnio.es/) [18]. The patient characteristics are summarized in Supplementary Tables 1, 2 and 3.

### Generation of NTRS

**Fig. 1:**
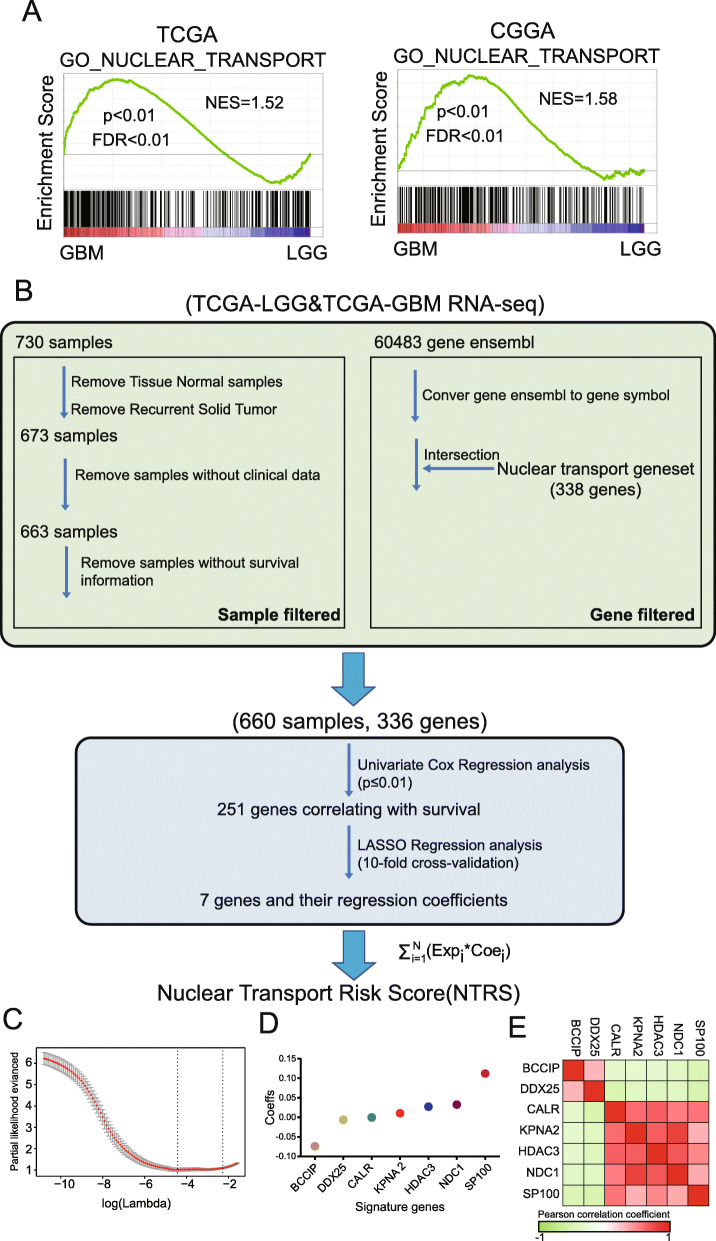
Identification of the 7-gene nuclear transport risk score (NTRS) via LASSO regression analysis in TCGA datasets. **a** Gene set enrichment analysis (GSEA) of nuclear transport between LGG and GBM in the training and validation datasets. NES: normalized enrichment score. **b** Development pipeline of NTRS. **c** Cross-validation with the TCGA dataset. **d** Coefficient values of the seven genes by LASSO. **e** Heatmap of Pearson correlation coefficient(r) of seven genes. Correlation between 7 genes was significant (*P* < 0.001)

The nuclear transport gene set was collected from the Molecular Signature Database v7.0 (http://software.broadinstitute.org/gsea/msigdb). Univariate Cox regression analysis was carried out to pre-filter genes associated with nuclear transport(*n* = 336) and 251 genes correlated with survival (*P* ≤ 0.01). Seven genes and their regression coefficients were calculated according to least absolute shrinkage and selection operator (LASSO) regression using the R package “glmnet “with parameters (family = “binomial”, type.measure = “deviance”, nfolds = 10) [14]. The risk score was calculated according to the formula presented in Fig. 1b.

### GSEA, enrichment map and GO clustering analysis

GSEA was performed with Gene Ontology (GO) biological process related gene sets (4436) (http://software.broadinstitute.org/gsea/msigdb/genesets.jsp?collection=BP) [19]. Enrichment map was used according to previously reported methods [20]. GO clustering analysis was performed using the R package “clusterProfiler”, in which the “enrichGO” and “dotplot” functions were employed to enrich genes and visualize gene clusters [21].

### Cell culture

The human glioblastoma cell line (LN-229) was obtained from ATCC and not passaged for more than half a year. Cells were cultured in DMEM (Gibco) containing 10% FBS and were incubated at 37 °C.

### Plasmid transfection and RT-PCR

The plasmids of NTRS related genes were obtained from Hanbio Inc. the transfection was performed according to the procedure of Lipofectamine 3000(Thermofisher). Three days after transfection, total RNA was extracted using RNAiso kit (Fastagen) and was reverse-transcribed (Takara). qRT-PCR was performed with SYBR Premix (Takara) using Bio-Rad RT-PCR System (CFX96). The results were normalized against GAPDH. The sequences of the primer were listed in Supplementary Table 4.

### Statistical analysis

The optimal cut-off value for NTRS was determined via ROC curve analysis. Briefly, in the ROC curves, the x-axis was plotted as “1-specificity” (false positivity), and the y-axis was plotted as the “sensitivity” (true positivity). The optimal cut-off value was determined on the basis of the Youden index (Y), which was the point with maximum sensitivity and specificity (Y = sensitivity+ specificity − 1) [22]. Student’s t test was performed to compare the NTRS values and relative expression of cell cycle genes of two different groups. Tukey’s multiple comparisons test was performed to compare the NTRS values of more than two groups. Differences in clinicopathological characteristics between groups were tested with chi-squared tests. Patient survival analysis was performed via the Kaplan-Meier method. Univariate and multivariate Cox regression analyses were performed to evaluate independent prognostic factors by using SPSS software. ROC curve analysis was performed to predict overall survival (OS). *P* < 0.05 was considered statistically significant. (*P < 0.05, **P < 0.01, ***P < 0.001).

## Results

### Identification of a 7-gene nuclear transport-related signature for the prognosis of glioma

**Fig. 2:**
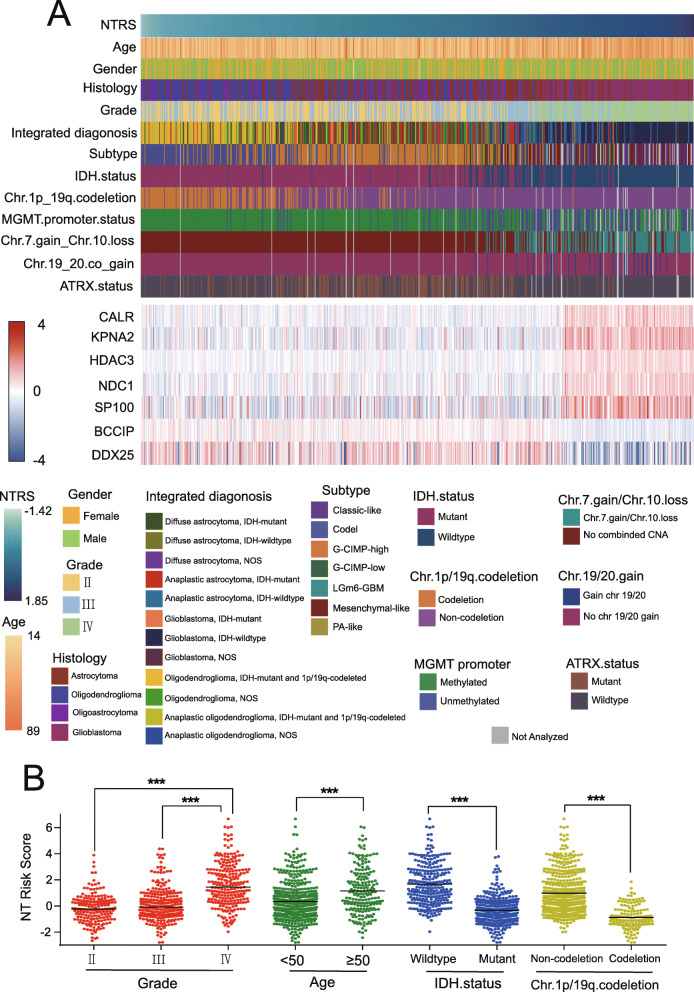
Association of NTRS and clinicopathological characteristics. **a** The distribution and association of NTRS and clinical or genetic characteristics in the training set (*n* = 660). **b** The distribution of NTRS in patients stratified by WHO grade, age, IDH status and 1p/19q status in the validation set. ^*^P < 0.05; ^**^P < 0.01; ^***^P < 0.001

First**,** we analyzed the expression of the nuclear transport gene set with the TCGA dataset. GBM showed distinct nuclear transport phenotypes from LGG (Supplementary Figure 1). Gene set enrichment analysis (GSEA) based on the TCGA and CGGA datasets also confirmed that the GBM group was enriched for transcriptional programs related to nuclear transport (Fig. 1a). To develop a gene signature based on the nuclear transport pathway, we first screened the glioma samples and nuclear transport-related genes in the training cohort. From the matrix of 660 gliomas and 336 genes (Supplementary Table 5), we selected 251 genes (Supplementary Table 6) associated with OS (*P* ≤ 0.01) by univariate Cox regression analysis (Fig. 1b). Seven genes were selected via LASSO regression analysis, and the nuclear transport risk score (NTRS) in the training cohort was obtained (Fig. 1c, d). Correlation between NTRS related seven genes were all significant (*P* < 0.001, Fig. 1e). Furthermore, BCCIP and DDX25 was decreased with grades, CALR, HDAC3, KPNA2, NDC1 and SP100 were increased with grades (Supplementary Figure 2). To analyze the relationships between NTRS and clinical characteristics, 660 patients from the training cohort and 668 patients from the validation cohort with clinical information were selected. The distribution of clinical characteristics, genetic characteristics and the expression of 7 genes in the patients are shown (Fig. 2a). As we expected, NTRS increased with glioma grade (Supplementary Figure 3A) and was higher in patients who were over 50 years old without IDH mutation or 1p/19q codeletion (Supplementary Figure 3B-D). Furthermore, in the subtype classified according to histology or molecular markers, NTRS was elevated in subgroups with shorter survival times, such as patients with the glioblastoma subtype or the subtype without IDH mutation and 1p/19q codeletion (Supplementary Figure 3E, F). These findings were validated in the CGGA dataset (Fig. 2b). In brief, NTRS was significantly associated with clinical and genetic characteristics that have been reported as prognostic markers in gliomas.

### Validity of NTRS as an independent prognostic marker in glioma

**Fig. 3:**
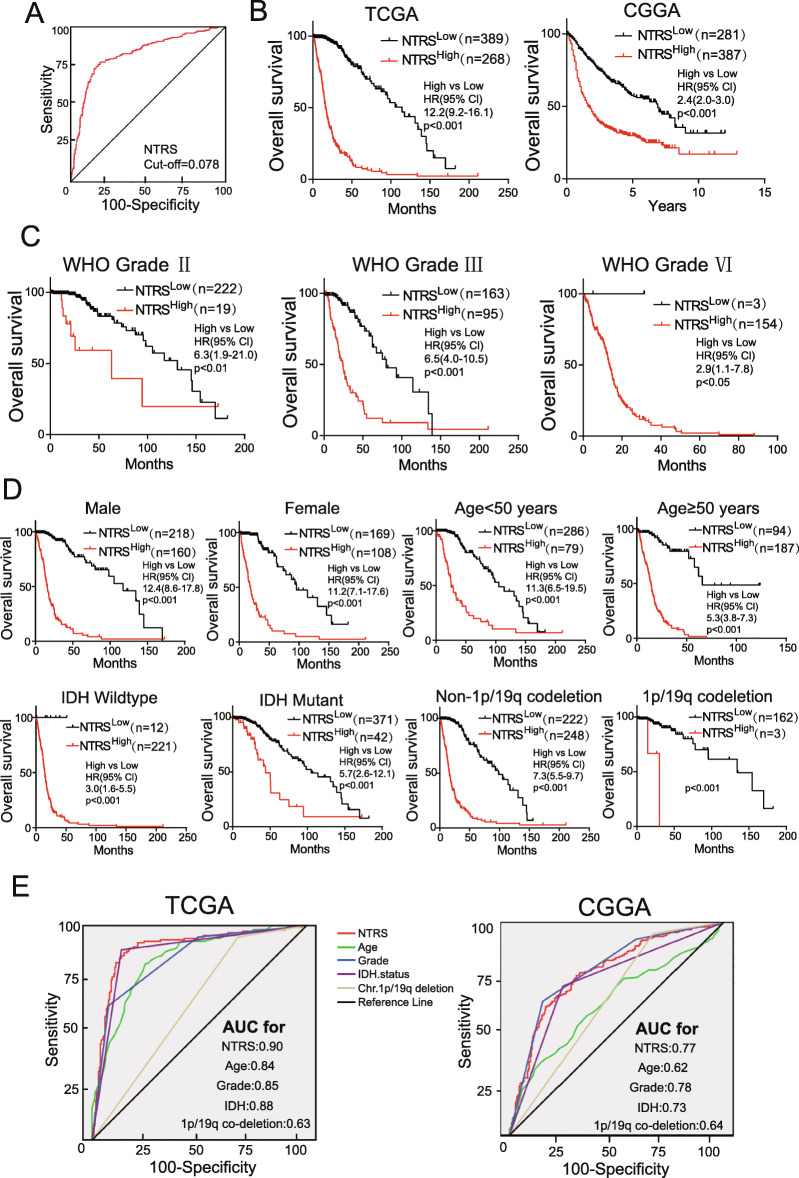
Prognostic significance of NTRS in glioma patients. **a** The cut-off value was determined by ROC analysis. Patients with a higher NTRS (> = 0.078) were classified as the NTRS^High^ group, and those with a lower NTRS (< 0.078) were classified as the NTRS^Low^ group. **b** survival analysis of glioma patients with a high NTRS (NTRS^High^) versus low NTRS (NTRS^Low^) in the training set and validation set. The hazard ratio was determined by the Mantel-Haenszel method, and the *P* value was determined by the chi-square test between the two groups. **c, d** Prognostic efficiency of NTRS in patients with different grades and subgroups. **e** ROC curves of the prediction of 2-year survival with NTRS and other markers in the training set and validation set

To investigate the prognostic value of NTRS, we first calculated the cut-off value by maximizing the Youden index through ROC analysis. The patients were divided into NTRS^High^ and NTRS^Low^ groups (Fig. 3a). Subsequently, we validated the correlation between the NTRS and clinicopathological factors in the TCGA dataset and CGGA dataset (Table 1). These data indicated that NTRS could be a potential prognostic marker for glioma. To test this hypothesis, we performed survival analysis in different cohorts and subgroups. Overall survival (OS) was significantly decreased in patients with high NTRS values compared to those with low NTRS values (hazard ratio 12.2, 95% confidence interval 9.2–16.1; *P* < 0.001, Fig. 3b, left panel). We also confirmed that in the validation cohort (hazard ratio 2.4, 95% confidence interval 2.0–3.0; *P* < 0.001, Fig. 3b, right panel). Furthermore, OS differed significantly between the NTRS^High^ and NTRS^Low^ groups in patients with gliomas of different grades, sexes, ages, IDH statuses and 1p/19q codeletion statuses (Fig. 3c, d). Through ROC analysis, we compared the sensitivity and specificity of NTRS with the traditional factors of age, grade, IDH status and 1p/19q codeletion status for the prediction of 2-year survival, revealing better predictive value of NTRS (Fig. 3e). These data indicated that NTRS is a promising prognostic marker for gliomas.

**Table 1:** Correlation between NTRS group and clinicopathological factors of glioma patients in the two cohorts

Features	Training set TCGA RNA-seq cohort (*n* = 660)			Validation set CGGA RNA-seq cohort (*n* = 668)		
	NTRS^Low^(*n* = 391)	NTRS^High^(*n* = 269)	*p*-value	NTRS^Low^(*n* = 281)	NTRS^High^(*n* = 387)	*p*-value
**Age**						
Mean (range)	41 (17–75)	56 (14–89)	< 0.001^***^	41 (12–69)	45 (11–76)	< 0.001^***^
**Gender**						
Female	170	108	0.38	135	153	< 0.05^*^
Male	219	161		146	234	
NA	2	0				
**WHO Grade**						
II	224	19	< 0.001^***^	106	74	< 0.001^***^
III	163	96		136	115	
IV	3	154		39	198	
NA	1	0				
**IDH status**						
Wildtype	12	221	< 0.001^***^	44	233	< 0.001^***^
Mutant	373	43		228	115	
NA	6	5		9	39	
**Chr.1p/19q status**						
Noncodeletion	223	249	< 0.001^***^	162	299	< 0.001^***^
Codeletion	163	3		117	24	
NA	5	17		2	64	
**Histology**						
Astrocytoma	120	71	< 0.001^***^	57	67	< 0.001^***^
Oligodendroglioma	168	17		52	29	
Oligoastrocytoma	100	27		133	93	
Glioblastoma	3	154		39	198	
**MGMT promoter status**						
Methylated	352	111	< 0.001^***^	/	/	
Unmethylated	30	123		/	/	
NA	9	35		/	/	
**Chr.7.gain&Chr.10.loss**						
Yes	1	146	< 0.001^***^	/	/	
No	380	106		/	/	
NA	10	17		/	/	
**Chr.19&20 gain**						
Non-gain	381	222	< 0.001^***^	/	/	
Gain	0	30		/	/	
NA	10	17		/	/	
**ATRX status**						
Wildtype	227	218	< 0.001^***^	/	/	
Mutant	155	33		/	/	
NA	9	18		/	/	

*P* < 0.05 (^*^), *P* < 0.01 (^**^) and *P* < 0.001 (^***^) is regarded as statistically significant

*NA* not applicable

Furtherly, we performed Cox regression analysis in the training set. In the univariate analysis, NTRS, age, histology, grade, IDH mutation, chromosome 1p/19q codeletion, MGMT promoter methylation, chromosome 9/10 status, ATRX mutation and chromosome 19/20 status were each associated with OS (*P* < 0.001). In the multivariable analysis, NTRS (hazard ratio 2.9, 95% confidence interval 1.74–4.82), age (hazard ratio 2.39, 95% confidence interval 1.66–3.45), grade (hazard ratio 1.99, 95% confidence interval 1.51–2.62), IDH status (hazard ratio 0.48, 95% confidence interval 0.29–0.80) and chromosome 19/20 status were independently associated with overall survival (Table 2). Accordingly, NTRS was validated as an independent prognostic marker in the CGGA cohort (Table 3). Taken together, these data indicated that NTRS could be an effective independent prognostic biomarker of gliomas.

**Table 2:** Cox regression analysis of overall survival related factors in the training cohort

Variable	Univariate analysis		Multivariable analysis	
	HR (95% CI)	*P*	HR (95% CI)	*P*
NTRS Group	8.84 (6.43–12.16)	< 0.001^***^	2.90 (1.74–4.82)	< 0.001^***^
Age	4.94 (3.63–6.72)	< 0.001^***^	2.39 (1.66–3.45)	< 0.001^***^
Gender	0.92 (0.70–1.22)	0.58	/	/
Histology	1.94 (1.66–2.25)	< 0.001^***^	/	/
Grade	4.86 (3.85–6.13)	< 0.001^***^	1.99 (1.51–2.62)	< 0.001^***^
IDH.status	0.10 (0.07–0.13)	< 0.001^***^	0.48 (0.29–0.80)	< 0.01^**^
Chr.1p/19q.codeletion	0.24 (0.15–0.38)	< 0.001^***^	/	/
MGMT.promoter.status	0.29 (0.22–0.39)	< 0.001^***^	/	/
Chr.7.gain&Chr.10.loss	8.75 (6.34–12.07)	< 0.001^***^	/	/
Chr.19&20 gain	3.37 (2.06–5.51)	< 0.001^***^	0.56 (0.34–0.93)	< 0.05^*^
ATRX.status	0.44 (0.32–0.62)	< 0.001^***^	/	/

NTRS (low and high); Gender (female and male); Histology (astrocytoma, oligodendroglioma, oligoastrocytoma and glioblastoma); Grade (II, III and IV); IDH status (wildtype and mutant); 1p/19q (non-codeletion and codeletion); MGMT promoter status (methylated and unmethylated); Chr.7.gain&Chr.10.loss (yes and no); Chr.19&20 gain (non-gain and gain); ATRX.status (wildtype and mutant)

*P* < 0.05 (^*^), *P* < 0.01 (^**^) and *P* < 0.001 (^***^) is regarded as statistically significant

*HR* hazard ratio, *CI* confidence interval

**Table 3:** Cox regression analysis of overall survival related factors in the validation cohort

Variable	Univariate analysis		Multivariable analysis	
	HR (95% CI)	*P*	HR (95% CI)	*P*
NTRS Group	3.01 (2.39-3.81)	< 0.001^***^	1.50 (1.14-1.98)	< 0.01^**^
Age	1.74 (1.39-2.19)	< 0.001^***^	/	/
Gender	0.95 (0.76-1.19)	0.67	/	/
Histology	1.51 (1.34-1.71)	< 0.001^***^	0.80 (0.69-0.93)	< 0.01^**^
Grade	2.81 (2.38-3.30)	< 0.001^***^	2.75 (2.13-3.55)	< 0.001^***^
IDH.status	0.32 (0.25-0.40)	< 0.001^***^	0.70 (0.53-0.92)	< 0.01^**^
Chr.1p/19q.codeletion	0.28 (0.20-0.39)	< 0.001^***^	0.55 (0.37-0.82)	< 0.01^**^

NTRS (low and high); Gender (female and male); Histology (astrocytoma, oligodendroglioma, oligoastrocytoma and glioblastoma); Grade (II, III and IV); IDH status (wildtype and mutant); 1p/19q (non-codeletion and codeletion); *P* < 0.05 (^*^), *P* < 0.01 (^**^) and *P* < 0.001 (^***^) is regarded as statistically significant

*HR* hazard ratio, *CI* confidence interval

### High NTRS gliomas exhibit accelerated cell cycle and enhanced immune responses

**Fig. 4:**
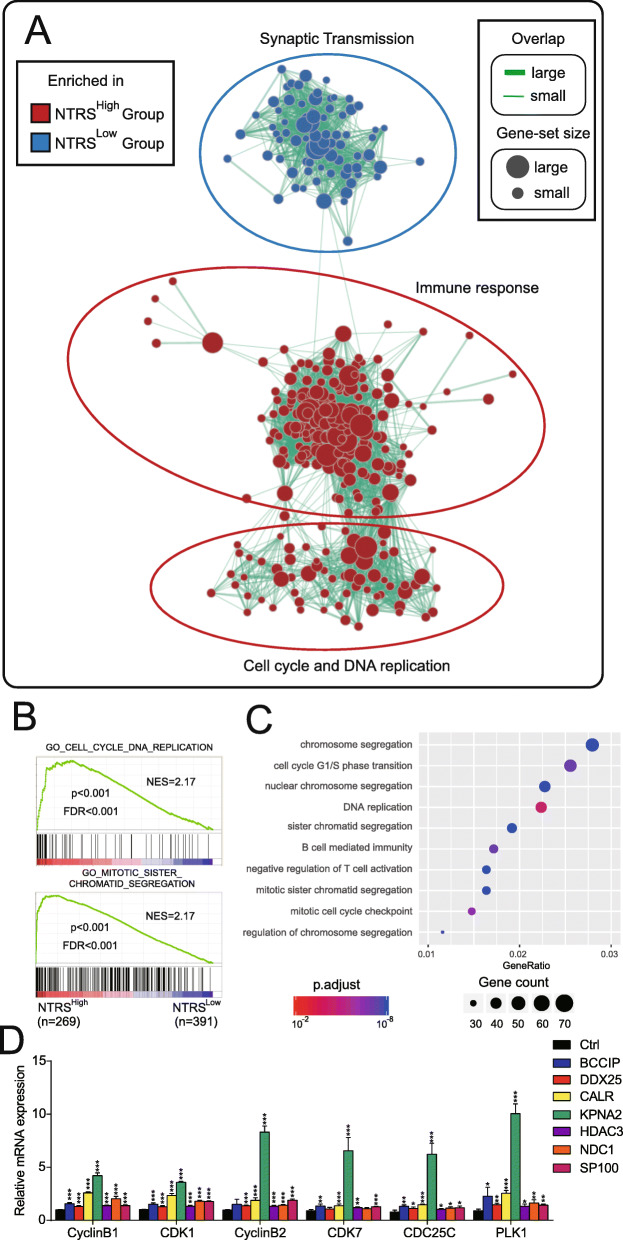
High NTRS gliomas exhibit accelerated cell cycle and enhanced immune responses. **a** Enrichment map of high NTRS group (*n* = 269) versus low NTRS group (*n* = 391). **b** Representative cell cycle related gene-sets in (**a**). **c** GO analysis of differentially expressed genes between low- and high-risk patients. **d** mRNA expression of cell cycle genes was detected in LN229 cells over-expressing indicated NTRS related genes

To analyze the association between NTRS and a poor prognosis of glioma patients, we performed GSEA and enrichment map analysis. The NTRS^High^ group was enriched in cell cycle and immune responses related gene-sets (Fig. 4a, b). Based on the identified differentially expressed genes (*P* < 0.05), GO analysis verified that the cell cycle and immune responses were significantly enriched in NTRS^High^ patients (Fig. 4c). Furthermore, we validated that the expression of cell cycle genes was significantly increased in glioma cells overexpressing NTRS related genes (Fig. 4d). These transcriptomic data indicated that NTRS^High^ gliomas exhibit accelerated cell cycle, which might result in a worse prognosis.

### NTRS is a potential marker for prognostic classification, combined with IDH mutation and 1p/19q codeletion

**Fig. 5:**
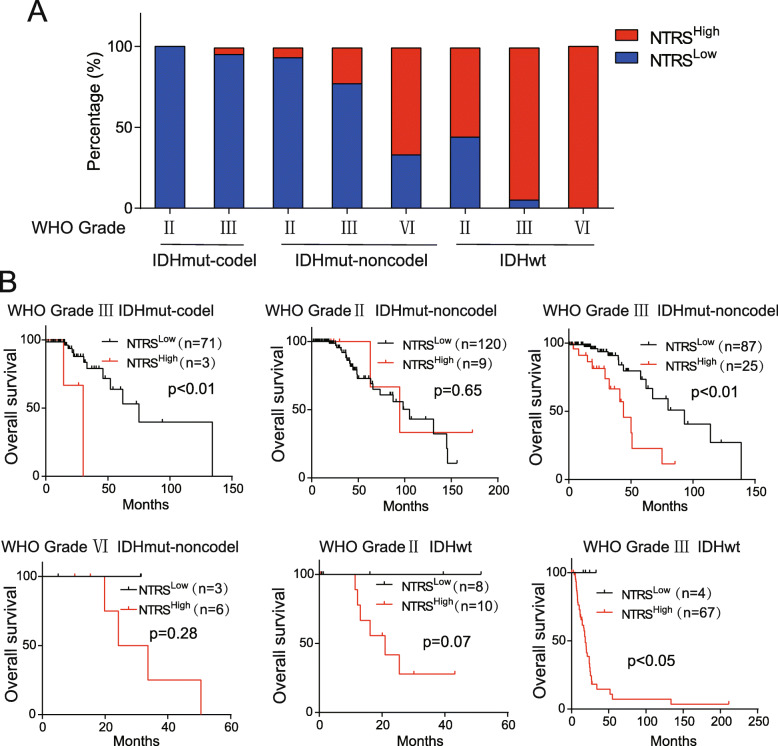
Prediction of prognosis with NTRS in cohorts stratified by WHO grade, IDH mutation and 1p/19q codeletion status. **a** Distribution of glioma patients with low and high NTRS in the indicated subgroups classified by WHO grade, IDH mutation and 1p/19q codeletion status. **b** Survival analysis was performed in glioma patients of (**a**) with a high NTRS versus low NTRS

**Fig. 6:**
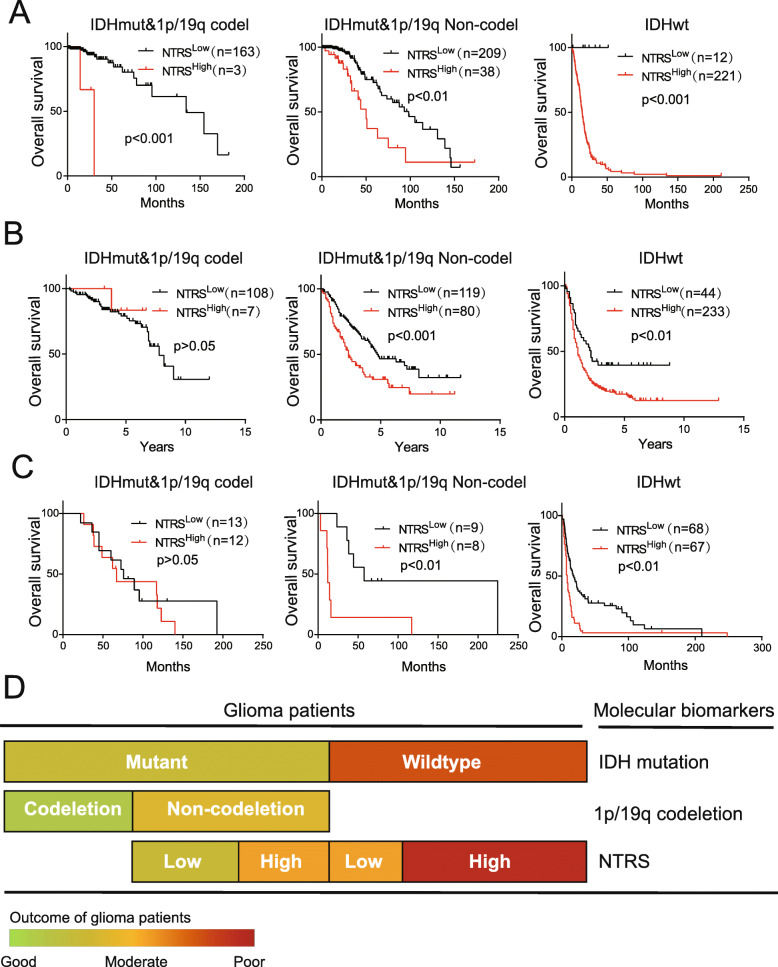
NTRS is a prognostic marker for molecular classification combined with IDH mutation and 1p/19q codeletion. **a, b** Overall survival analysis of glioma patients with the indicated mutations in the training set (TCGA for **a**) and validation sets (CGGA for **b** and Grevendeel for **c**). **d** Proposed prognostic classification for glioma combining IDH mutation, 1p/19q codeletion and NTRS. The variation in color from green to red represents the patients’ outcome from good to poor

To illustrate the value of NTRS in the classification of gliomas, we first analyzed the distribution of subtypes stratified by WHO grade, IDH mutation and 1p/19q codeletion status in the NTRS group. In gliomas with IDH mutation and 1p/19q codeletion, all gliomas diagnosed as WHO grade II (100%, 92/92) were associated with a low NTRS, whereas only 4% of gliomas diagnosed as WHO grade III (3/74) were associated with a low NTRS. In gliomas with IDH mutations and without 1p/19q codeletion, the rate of high NTRS values increased according to the WHO grade (7%, 9/129 for grade II; 22%, 25/112 for grade III; 66%, 4/6 for grade IV). In gliomas without IDH mutations, 56% of gliomas diagnosed as WHO grade II (10/18), 94% as WHO grade III (68/72) and 100% as WHO grade IV (143/143) exhibited a high NTRS (Fig. 5a). Subsequently, we performed survival analysis in different subgroups. The NTRS^High^ group exhibited shorter survival among patients with WHO grade III gliomas classified by IDH mutation and 1p/19q codeletion (Fig. 5b). These results indicated that NTRS could be more effective as a marker when combined with other prognostic markers for gliomas. To test this hypothesis, we analyzed the prognostic value in subgroups stratified by IDH mutation and 1p/19q codeletion. In both the subgroup with IDH mutation without 1p/19q codeletion and the subgroup without IDH mutation, overall survival (OS) was decreased in patients with a high NTRS (Fig. 6a). These results were confirmed in the validation cohort (Fig. 6b). Furthermore, we established NTRS with microarray data of Rembrandt, Grevendeel and Kamoun cohorts. As same as RNA-seq cohorts, The NTRS^High^ group exhibited shorter survival (Supplementary Figure 4). Patients with high NTRS exhibited worse prognosis in subgroup of IDH mutation only and subgroup without IDH mutation or 1p/19q codeletion (Fig. 6c). In conclusion, by combining data on IDH mutation and 1p/19q codeletion with NTRS, we established a prognostic classification model for survival prediction in glioma patients (Fig. 6d).

## Discussion

The nuclear transport system has been proven to be critical for tumorigenesis and the development of cancer [7]. Nuclear transport could serve as a therapeutic target in several cancer types [5, 23, 24]. Many genes involved in nuclear transport have been reported to be associated with the prognosis of cancer patients [25]. These results indicate that nuclear transport may serve as a marker of prognosis in cancer. In this study, we used RNA-seq data from the TCGA and CGGA databases to generate a seven-gene nuclear transport risk score (NTRS) to predict the prognosis of glioma patients. We confirmed that NTRS was an independent prognostic marker and better predicted overall survival compared to traditional factors. Our work establishes a novel nuclear transport-based gene signature for the prediction of glioma patient survival. Furthermore, In the seven genes, several are involved initiation and progression of gliomas [26–28]. Especially, KPNA2 could interact with nuclear localization signal (NLS)-containing cargoes and is involved in the nuclear transport of proteins such as TP53, E2F1 and c-myc [29, 30]. These data indicated that NTRS related genes are not just prognostic marker, but also play essential role in proliferation and invasion of gliomas.

One shortcoming of this work was the lack of clinical validation and functional research on NTRS. With the development of RT-PCR, Nanostring and next-generation sequencing (NGS), gene signatures have been broadly applied in the clinic for the prediction of recurrence and the response to therapy [31–33]. Gene signature panels based on NTRS should be developed, and real-world research (RWR) involving multiple centers should be performed in the future. Although all seven genes were significantly associated with survival in multiple datasets (TCGA, CGGA and Rembrandt) and several of the genes have been reported to be functional in gliomas [26, 30, 34], further experiments are needed to study the function and mechanism of these seven genes, which will be performed in the future.

Since the publication of the 2016 WHO classification of tumors of the central nervous system, integrated classification has been generally applied to glioma. With the availability of public databases, the integration of data on histology and mutation, methylation and mutation or mRNA expression and mutation can divide patients into different subgroups [12, 13, 35, 36]. Furthermore, with the development of artificial intelligence and machine learning, digital images obtained via magnetic resonance imaging and histopathological analysis can be used to predict not only overall survival but also IDH mutation and 1p/19q codeletion [37, 38]. In the near future, the diagnosis of gliomas will involve the combination of multidimensional data. At the molecular level, glioma panels including mutation, methylation and gene expression data will be rapidly developed. In this study, we made a preliminary attempt to combine NTRS with IDH mutation and 1p/19q codeletion data for prognosis. Patients with high NTRS exhibited worse prognosis in subgroup of IDH mutation only and subgroup without IDH mutation or 1p/19q codeletion. The patients in the five subgroups exhibited significantly different outcomes (Fig. 6d). Our research demonstrated that the nuclear transport-related gene signature could serve as a novel marker for prognostic classification in combination with IDH mutation and 1p/19q codeletion.

## Conclusions

Risk score based on nuclear transport system is significantly associated with poor clinicopathologic characteristics and is an independent prognostic marker in diffuse gliomas.

The nuclear transport risk score combined with IDH mutation and 1p/19q codeletion could better predict the overall survival of glioma patients.

## Supplementary Information

**Additional file 1: Supplemental Figure 1.** Heatmap of nuclear transport genes in lower-grade gliomas and glioblastomas.

**Additional file 2: Supplemental Figure 2.** FPKM value of NTRS related seven genes in patients stratified by WHO grade.

**Additional file 3: Supplemental Figure 3.** Distribution of NTRS in patients stratified by WHO grade (A), age (B), IDH status (C), 1p/19q status (D), histology (E) and molecular subtype (F) in the training set. ^*^P < 0.05; ^**^P < 0.01; ^***^P < 0.001.

**Additional file 4: Supplemental Figure 4.** overall survival analysis of glioma patients with a high NTRS (NTRS^High^) versus low NTRS (NTRS^Low^) in Rembrandt, Grevendeel and Kamoun dataset. Median of NTRS as cut-off value.

**Additional file 5: Supplemental Table 1.** The clinicopathological characteristics of the glioma patients enrolled in this study.

**Additional file 6: Supplemental Table 2.** Clinical characteristics and NTRS groups of the training cohort (*n* = 660).

**Additional file 7: Supplemental Table 3.** Clinicopathological characteristics and NTRS groups of the validation cohort (*n* = 668).

**Additional file 8: Supplemental Table 4.** The sequences of the primers.

**Additional file 9: Supplemental Table 5.** The list of 336 nuclear transport related genes.

**Additional file 10: Supplemental Table 6.** The list of 251 genes filtered by Univariate Cox regression analysis.

## Data Availability

The datasets generated and/or analyzed during the current study are available in the TCGA (http://www.cbioportal.org) and CGGA (http://gliovis.bioinfo.cnio.es/) databases.

## Abbreviations

NTRS: Nuclear transport risk score
FBS: Fetal bovine serum
LASSO: Least absolute shrinkage and selection operator
NPC: Nuclear pore complex
WHO: World health organization
GSEA: Gene set enrichment analysis
TCGA: The Cancer Genome Atlas
CGGA: Chinese Glioma Genome Atlas
GSEA: Gene Set Enrichment Analysis
GBMs: Glioblastomas
LGGs: Lower-grade gliomas
IDH: Isocitrate dehydrogenase
ROC: Receiver operating characteristics
AUC: Area under curve
GO: Gene Ontology
BP: Biological process
OS: Overall survival
NGS: Next-generation sequencing
FPKM: Fragments per kilobase per million
