# Supplementary figures and images for: A nuclear transport-related gene signature combined with IDH mutation and 1p/19q codeletion better predicts the prognosis of glioma patients
Source: BMC Cancer. 2020 Nov 9;20:1072. doi: 10.1186/s12885-020-07552-3 (PMC7654069; doi:10.1186/s12885-020-07552-3)

Nuclear transport genes(n=336)

Glioblastomas(n=157)

Lower grade gliomas(n=503)

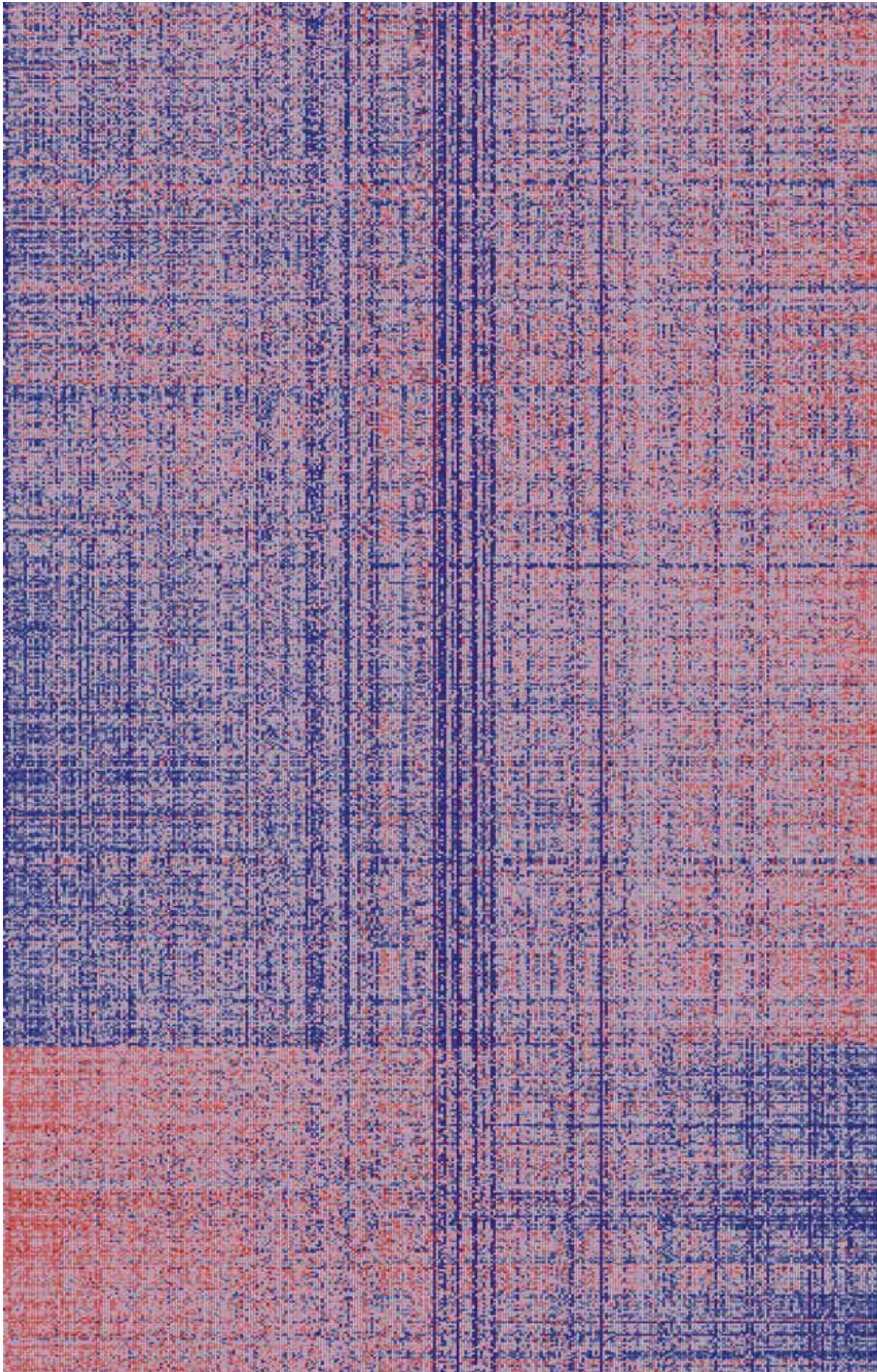

Supplement: Supplementary file 1 — Additional file 1: Supplemental Figure 1. Heatmap of nuclear transport genes in lower-grade gliomas and glioblastomas. [file 12885_2020_7552_MOESM1_ESM.pdf]

BCCIP

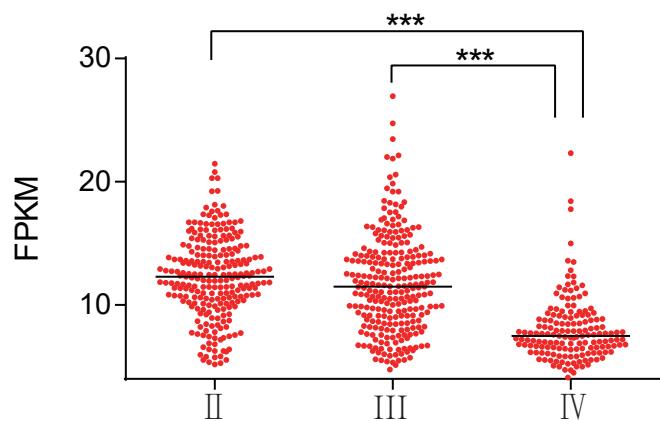

DDX25

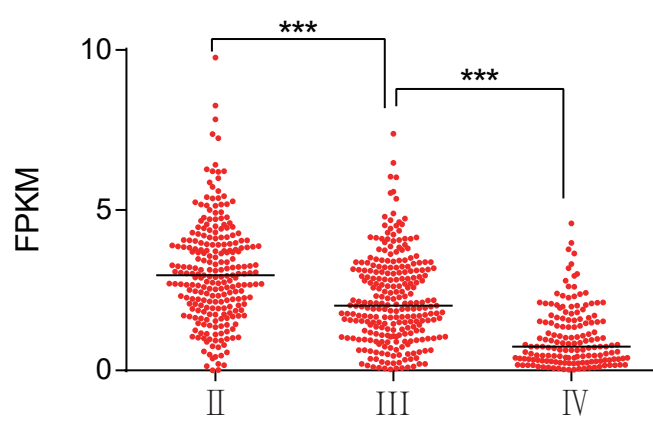

CALR

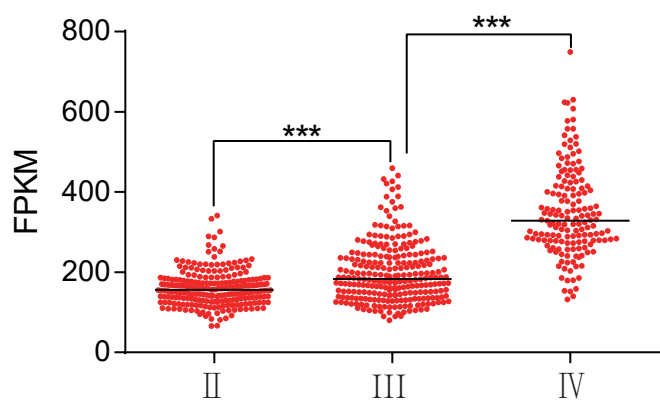

HDAC3

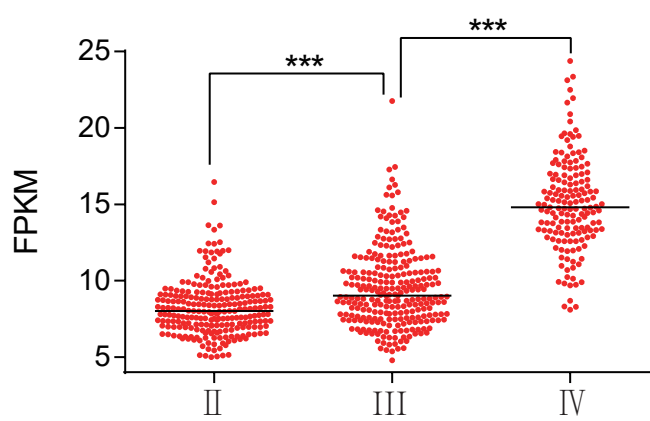

KPNA2

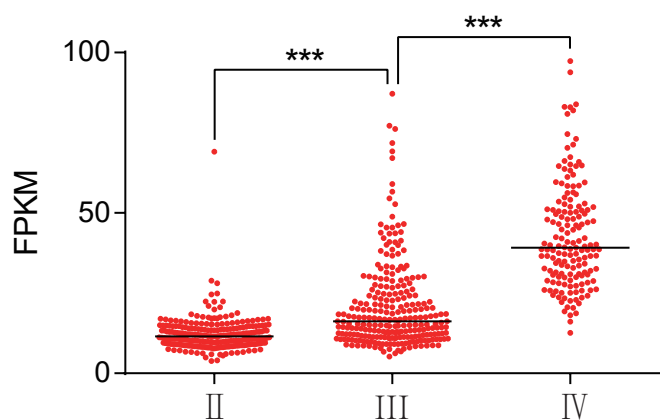

NDC1

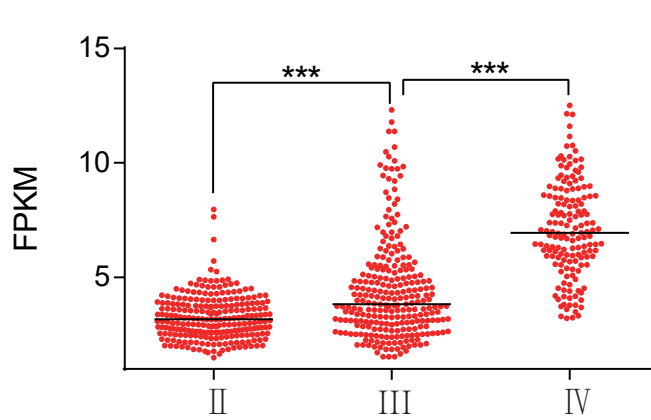

SP100

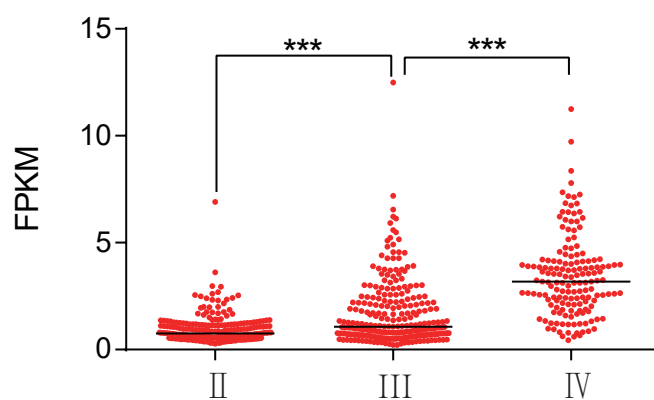

Supplement: Supplementary file 2 — Additional file 2: Supplemental Figure 2. FPKM value of NTRS related seven genes in patients stratified by WHO grade. [file 12885_2020_7552_MOESM2_ESM.pdf]

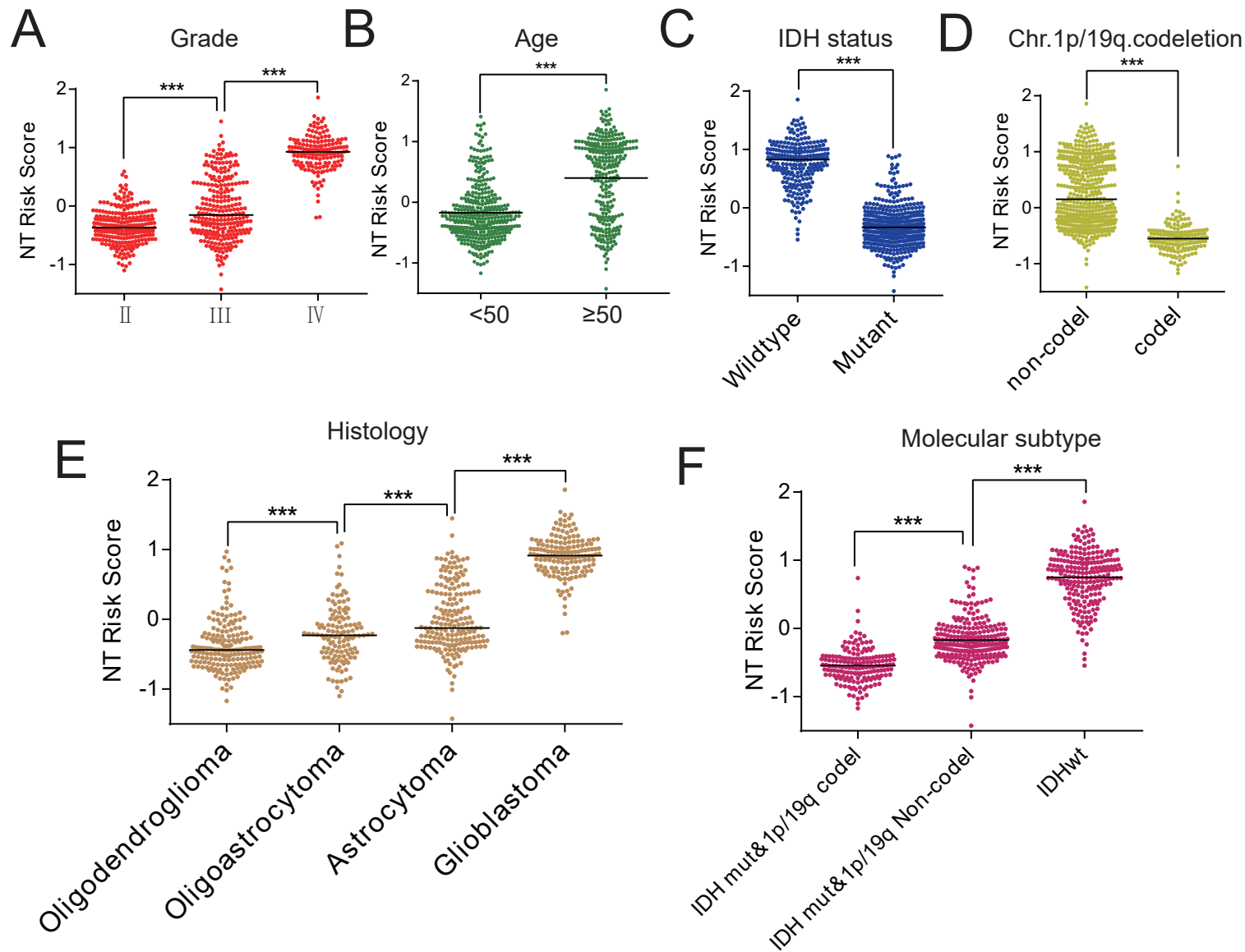

Supplement: Supplementary file 3 — Additional file 3: Supplemental Figure 3. Distribution of NTRS in patients stratified by WHO grade (A), age (B), IDH status (C), 1p/19q status (D), histology (E) and molecular subtype (F) in the training set. *P < 0.05; **P < 0.01; ***P < 0.001. [file 12885_2020_7552_MOESM3_ESM.pdf]

Rembrandt

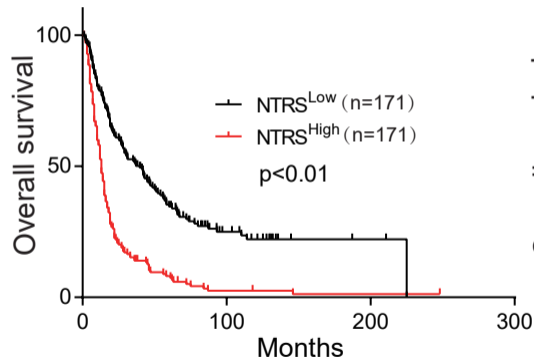

grevendeel

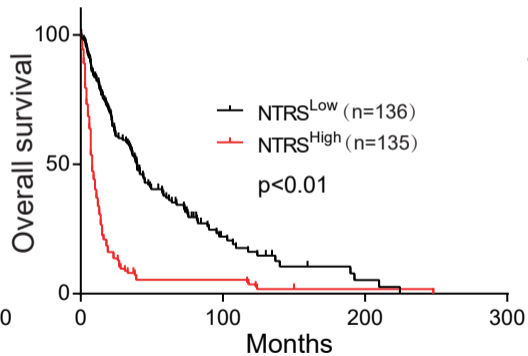

Kamoun

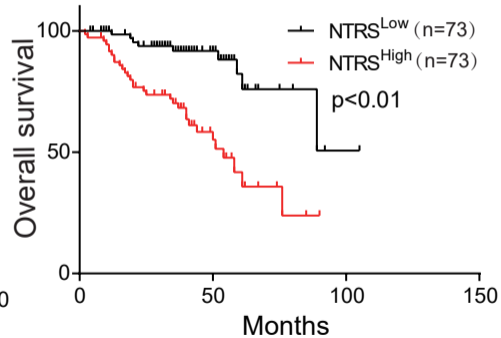

Supplement: Supplementary file 4 — Additional file 4: Supplemental Figure 4. overall survival analysis of glioma patients with a high NTRS (NTRSHigh) versus low NTRS (NTRSLow) in Rembrandt, Grevendeel and Kamoun dataset. Median of NTRS as cut-off value. [file 12885_2020_7552_MOESM4_ESM.pdf]
