# Supplementary material for: A nuclear transport-related gene signature combined with IDH mutation and 1p/19q codeletion better predicts the prognosis of glioma patients
Source: BMC Cancer. 2020 Nov 9;20:1072. doi: 10.1186/s12885-020-07552-3 (PMC7654069; doi:10.1186/s12885-020-07552-3)
Supplement: Supplementary file 5 — Additional file 5: Supplemental Table 1. The clinicopathological characteristics of the glioma patients enrolled in this study. [file 12885_2020_7552_MOESM5_ESM.docx]

supplementary table 1. The clinicopathological characteristics of the glioma patients enrolled in this study.

| **Characteristics** | **Training set (TCGA)** | **Validation set (CGGA)** |
| --- | --- | --- |
| **Age (year)** | | |
| Mean (range) | 47（14-89） | 43（11-76） |
| **Gender** | | |
| Female | 278 | 288 |
| Male | 380 | 380 |
| NA | 2 | 0 |
| **Histology** | | |
| Astrocytoma | 191 | 124 |
| Oligodendroglioma | 185 | 81 |
| Oligoastrocytoma | 127 | 226 |
| Glioblastoma | 157 | 237 |
| **WHO Grade** | | |
| Ⅱ | 243 | 180 |
| Ⅲ | 259 | 251 |
| Ⅳ | 157 | 237 |
| NA | 1 | NA |
| **IDH status** | | |
| Wildtype | 233 | 277 |
| Mutant | 416 | 343 |
| NA | 11 | 48 |
| **Chr.1p/19q** | | |
| Non-codeletion | 472 | 461 |
| Codeletion | 166 | 141 |
| NA | 22 | 66 |
| **MGMT promoter status** | | |
| Methylated | 463 | / |
| Unmethylated | 153 | / |
| NA | 44 | / |
| **Chr.7.gain&Chr.10.loss** | | |
| Yes | 147 | / |
| No | 486 | / |
| NA | 27 | / |
| **Chr.19&20 gain** | | |
| Non-gain | 603 | / |
| Gain | 30 | / |
| NA | 27 | / |
| **ATRX status** | | |
| Wildtype | 445 | / |
| Mutant | 188 | / |
| NA | 27 | / |
